# Conjugated polymers mediate effective activation of the Mammalian Ion Channel Transient Receptor Potential Vanilloid 1

**DOI:** 10.1038/s41598-017-08541-6

**Published:** 2017-08-16

**Authors:** F. Lodola, N. Martino, G. Tullii, G. Lanzani, M. R. Antognazza

**Affiliations:** 1 Center for Nano Science and Technology, IIT@PoliMi, via Pascoli 70/3, 20133 Milano, Italy; 2 Politecnico di Milano, Dipartimento di Fisica, Piazza L. Da Vinci 32, 20133 Milano, Italy; 3 Wellman Center for Photomedicine, Massachusetts General Hospital and Harvard Medical School, Cambridge, Massachusetts 02139 USA

## Abstract

Selective and rapid regulation of ionic channels is pivotal to the understanding of physiological processes and has a crucial impact in developing novel therapeutic strategies. Transient Receptor Potential (TRP) channels are emerging as essential cellular switches that allow animals to respond to their environment. In particular, the Vanilloid Receptor 1 (TRPV1), besides being involved in the body temperature regulation and in the response to pain, has important roles in several neuronal functions, as cytoskeleton dynamics, injured neurons regeneration, synaptic plasticity. Currently available tools to modulate TRPV1 activity suffer from limited spatial selectivity, do not allow for temporally precise control, and are usually not reversible, thus limiting their application potential. The use of optical excitation would allow for overcoming all these limitations. Here, we propose a novel strategy, based on the use of light-sensitive, conjugated polymers. We demonstrate that illumination of a polymer thin film leads to reliable, robust and temporally precise control of TRPV1 channels. Interestingly, the activation of the channel is due to the combination of two different, locally confined effects, namely the release of thermal energy from the polymer surface and the variation of the local ionic concentration at the cell/polymer interface, both mediated by the polymer photoexcitation.

## Introduction

Cellular functions are primarily mediated by ionic channels, a class of integral membrane proteins found in all cells of the body. Selective and rapid regulation of specific channels is key to the understanding of physiological processes, the development of novel therapeutic strategies and the implementation of specific drugs^1–3^. One of the largest groups of ion channels is represented by the Transient Receptor Potential (TRP) channels superfamily, which has been very intensively investigated in recent years^4, 5^. TRPs are widespread in eukaryotes, from yeast to mammals, they are characterized by remarkable diversity in ionic selectivity and activation mechanisms, and they are sensitive to a wide range of chemical and physical stimuli^6^. Importantly, they have many functional roles in both excitable and non-excitable cells, some of which are still to be completely elucidated. In more detail, TRPs behave as essential cellular switches that allow organisms to respond to a variety of environmental stimuli, and are at the base of the perception of pain, warm and cold temperatures, noxious and pungent chemicals, and pressure^6^. The Vanilloid Receptor 1 (TRPV1) is by far the most studied channel belonging to the TRP super family. Besides being involved in the regulation of the body temperature^7^ and in the response to painful stimuli^8^, it also has important functional roles in the neurogenic inflammatory response^9^. It has been demonstrated that upon nerve injury, changes in TRPV1 expression occur in damaged nerve fibers and cell bodies within sensory ganglia, leading to enhanced spontaneous excitation^10^. TRPV1 is also believed to be involved in forms of synaptic remodeling^11^, to potentiate glutamatergic signaling^12^ and to contribute to cytoskeletal remodeling^13^. Notably, the expression of TRPV1 channels in retinal ganglion cells positively influences their survival in the presence of optical neuropathies, stress events, such as ischemia, and neurodegenerative diseases^14–16^.

Overall, the ensemble of the recent experimental evidences justifies the high interest for both activating and inhibiting agents of TRPV1, as novel physiological probes and powerful pharmacological targets. Both TRPV1-antagonist and -agonist therapies are under intense investigation^17–21^. TRPV1 can be activated by several chemical/physical stimuli, including voltage^22^, heat^23^, chemical compounds such as capsaicin^24^ and spider toxins^25^, acid pH^8, 26^, several fatty acids such as the endocannabinoid anandamide^27^, or by some of their possible combinations. Most common activation protocols, relying on the application of a transmembrane potential, administration of capsaicin to the cell medium, and heating of the extracellular bath above the threshold temperature of 43 °C, all suffer from limited temporal and spatial resolution and are often irreversible, thus requiring frequent washing outs of the cell media. Use of optical excitation would allow for overcoming the limitations of existing methods and gaining spatially selective, temporally precise and reversible control of TRPV1 channel activity. Very recently, two different, light activated methods have been proposed, based on the use either of photoswitchable compounds^28^, composed by capsaicin derivatives linked to azobenzene molecules, or of light sensitive nanoparticles with high photothermal conversion efficiency^29^. Both of them exploited the synergistic combination of visible light with specific stimuli, fatty acids activators and heat, respectively.

Here, we propose an alternative strategy, based on the use of light sensitive, conjugated polymers thin films. We demonstrate that illumination of the polymer leads to reliable, robust and temporally precise control of TRPV1 channel activity. Interestingly, the activation of the channel can be interpreted on the base of the combined action of two different effects, both of them localized at the polymer surface: the release of thermal energy from the excited photoactive material, and the variation of the ionic concentration in the cleft niche, mediated by photoexcitation and accumulation of photo-generated negative charges at the interface between the polymer and the extracellular bath.

## Results and Discussion

### Realization of the bio/polymer interface

**Figure 1:**
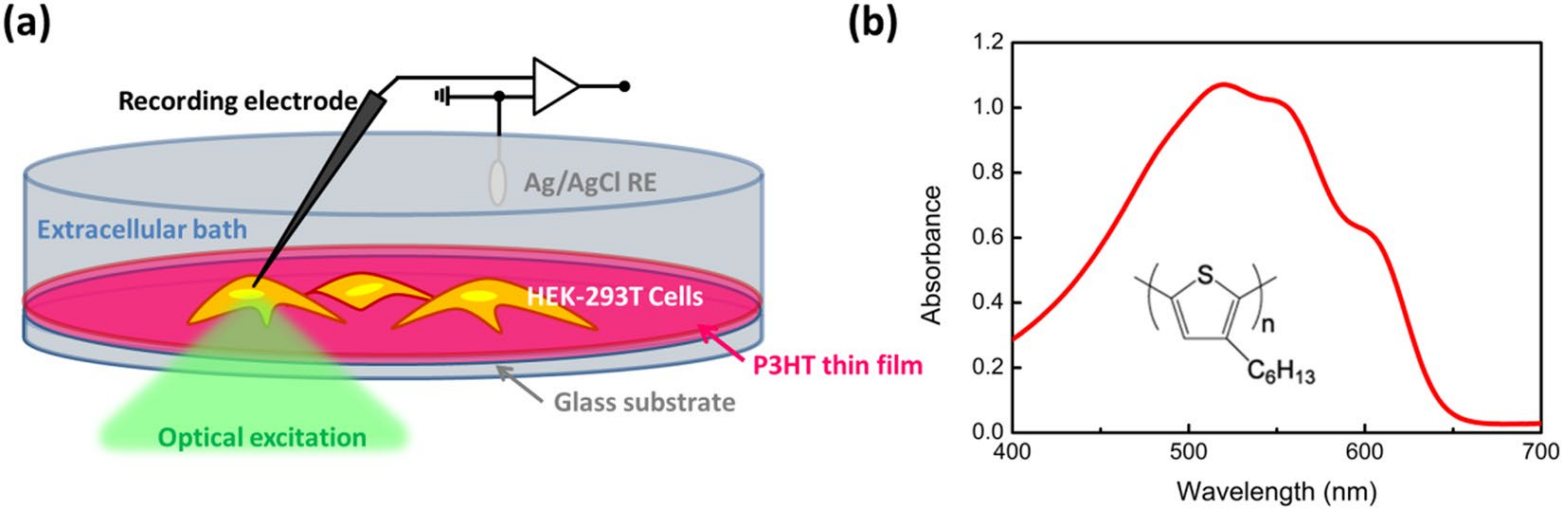
Sketch of the experimental set-up for optical stimulation of living cells. (**a**) HEK-293T cells, stably transfected with the human TRPV1 channel, are grown directly on top of the polymer surface. Green light incident from the bottom side is used to optically excite the conjugated polymer. Cell electrical activity is recorded by the patch clamp technique, in whole-cell configuration. (**b**) rr-P3HT chemical structure and optical absorption of the thin film.

Figure 1a shows a sketch of the polymer/cell interface developed for activation of TRPV1 channels. A thin film (approximate thickness, 250 nm) of regio-regular poly-3(hexylthiophene) (P3HT) is deposited by spin coating on top of a glass substrate. P3HT is characterized by a wide optical absorption spectrum peaking at 520 nm (Fig. 1b), excellent optoelectronic properties when used as the active, light absorbing and charge generating material in solar cells and organic photodetectors^30^ and outstanding biocompatibility properties when used in combination with living cells, as recently demonstrated in both *in vitro* and *in vivo* studies^31–34^. Moreover, P3HT was recently reported to efficiently sustain thermally-mediated depolarization and hyperpolarization effects in a number of different biological systems, including primary hippocampal neurons, excised retinal tissues and epileptic brain slices^35^. As the biological counterpart of the bio/polymer interface, we use Human Embryonic Kidney (HEK-293) cells stably transfected with the human TRPV1 channel (HEK-293T). HEK-293T cells were cultured directly on top of the polymer surface, as well as on control glass substrates, for 48 h. Both polymer and glass substrates were pre-treated by deposition of a fibronectin adhesion layer. The tetrazolium salt (MTT) assay has been used to obtain a quantitative colorimetric evaluation of cell survival and proliferation up to 4 days *in vitro*. Cells cultured on top of the polymer surface show good viability properties, even though their proliferation rate is slightly slowed down respect to control glass substrates (Figure S1). Polymer photoexcitation is provided by the emission of a green led light source (emission peak, 544 nm; photoexcitation density in the range 26.1–343.9 mW/mm^2^), impinging on the polymer sample from the glass side. It is important to notice that, due to the high absorbance of the polymer layer, the actual optical power density impinging on the cultured cells is reduced and falls within the range 3.48–86.96 mW/mm^2^ (see Methods section for details).

We preliminarily verified the presence and functional excitability of the human TRPV1 channel in cellular networks cultured on glass substrates by carrying out whole-cell patch clamp experiments in the presence of Capsaicin (50 nM), one of the most powerful TRPV1 activators^23^. In good agreement with previous reports^23, 36^, a robust increase of the inward and outward currents is observed, thus confirming the presence of TRPV1 channels in the employed stable line (Figure S2). On the other side, non-transfected HEK-293 cells did not show any significant response to the pharmacological stimulus (data not shown).

### Polymer-mediated optical modulation of the TRPV1 channel

**Figure 2:**
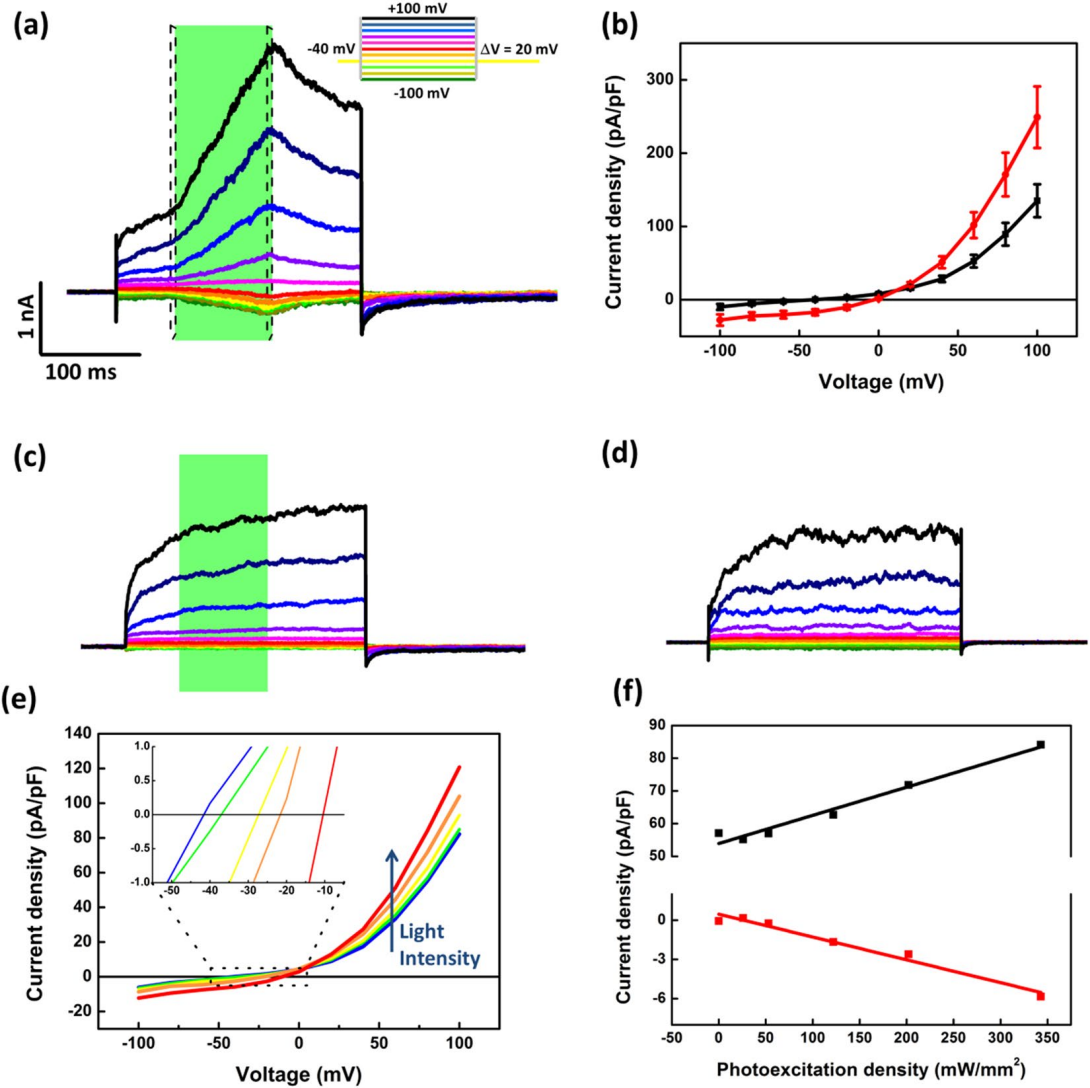
Outward current increase in HEK-293T cells upon photostimulation with 100 ms light pulses. (**a**) Representative whole-cell current traces in response to voltage steps from −100 mV to +100 mV. Holding potential, −40 mV (protocol in the inset). Photoexcitation is represented by the green shaded area. Light power density, 202.4 mW/mm^2^. (**b**) Current–voltage characteristics acquired in cells immediately before the light onset (black line) and upon 343.9 mW/mm^2^ photoexcitation density (red line). Data have been calculated as an average over the 5 ms-wide temporal windows represented in panel (**a**) as dashed rectangles. (**c**,**d**) Control measurements in cells grown on glass (**c**) and on P3HT thin films not subjected to optical excitation (**d**,**e**) Current voltage characteristics recorded under different photoexcitation densities (26.1 mW/mm^2^, blue solid line; 53 mW/mm^2^, green solid line; 122.6 mW/mm^2^, yellow solid line; 202.4 mW/mm^2^, orange solid line; 343.9 mW/mm^2^, red solid line). Data have been calculated as the average values recorded during the whole temporal window of the illumination protocol (green rectangle in panel (**a**,**f**) Current density values show a linear dependence on light photoexcitation density. Red and black symbols refer to two representative data sets at fixed potential, + 80 mV and −40 mV, respectively. Solid lines display the linear regression fitting. R^2^ = 0.97 in both cases. All experiments were carried out at room temperature (24 °C).

The effect of polymer-mediated optical stimulation on the excitability of HEK-293T cells was first assessed by voltage-clamp whole-cell recordings. Visible light pulses (wavelength excitation peak, 544 nm; photoexcitation density, 202.4 mW/mm^2^) lead to a remarkable increase of the outward current (Fig. 2a and Figure S3a for 100 ms- and 20 ms- light stimuli duration, respectively). Figures 2b and S3c report the average current increase due to the photoexcitation, as obtained by averaging current values immediately before the light onset (in black), and immediately before the light offset, where the maximum current is recorded (in red), over 5-ms temporal windows (represented in panel a as dashed lines). In particular, at the maximum applied voltage (+100 mV), the positive variation of the current amounts at +84% and +26%, for 100 ms- and 20 ms- long pulses, respectively. We notice that the response amplitude (249 ± 41 pA/pF for 100 ms pulses) is comparable to the one obtained upon capsaicin administration at 50 nM concentration (224 ± 27 pA/pF, Figure S2), which is considered to be of physiological significance for pharmacological treatments^23^. Control measurements were carried out both on HEK-293T cells cultured on glass substrates and subjected to the same illumination protocols (Fig. 2c and Figure S3b), and on HEK-293T cultured on polymer substrates, but kept in dark conditions (Fig. 2d). In both cases no significant signal variation is recorded, thus indicating that the effect observed in cells cultured onto polymers is due to an optically-mediated process. I–V characteristics (Fig. 2e) upon optical stimulation at different photoexcitation densities, varying within the range 26.1 mW/mm^2^ − 343.9 mW/mm^2^, show that the reversal potential (defined as the value of the potential at which the current density changes its sign) shifts towards more positive values at increasing light intensities (see inset), from −41 mV at 26.1 mW/mm^2^ up to −10.4 mV at 343.9 mW/mm^2^, consistently with the possible activation of cationic channels. A linear dependence of the current from the light photoexcitation density is observed, at both positive and negative voltages (see Fig. 2f for two representative examples, at +80 mV and −40 mV).

Importantly, non-transfected cells show a markedly different behavior, displaying a current increase upon light stimulation lower than one order of magnitude (Figure S4). Altogether, these data first indicate that the recorded signal may be attributed to the excitation of the TRPV1 channel.

**Figure 3:**
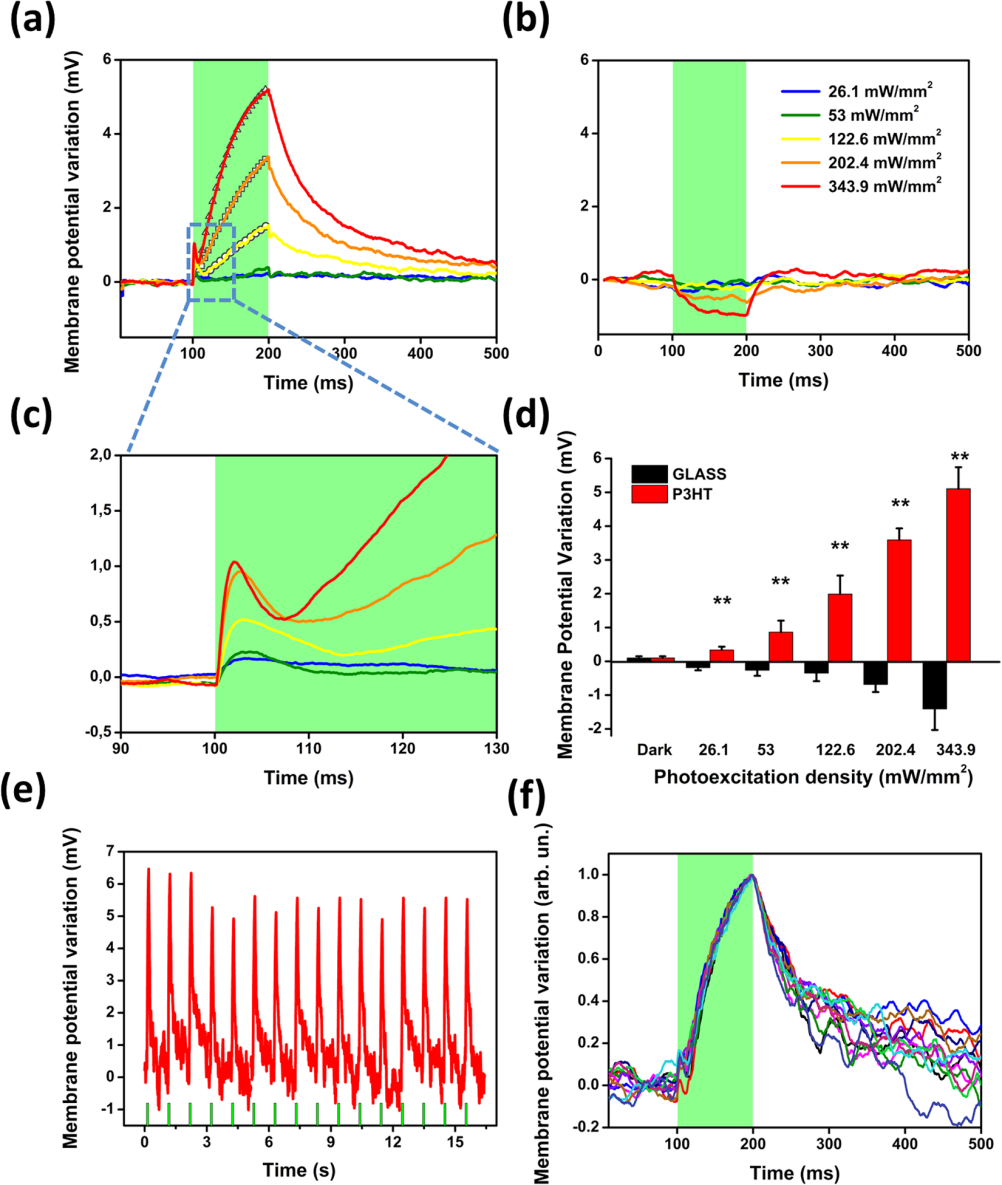
HEK-293T cells depolarization mediated by polymer optical excitation. Membrane potential variation measured in HEK-293 cells stably expressing human TRPV1 seeded on P3HT (**a**,**c**) and bare glass (**b**) when photostimulated with 100 ms visible light pulses, represented by green shaded areas, at increasing photoexcitation densities (from 26.09 to 343.91 mW/mm^2^). Every trace is obtained as the average of 40 consecutive sweeps. The experiments are performed at 24 °C. Mean values ± MSE of the photoinduced membrane potential variation are reported in panel (**d**), n = 10 cells each condition. Asterisks indicate statistical significance (Student’s t-test, **p < 0.001). (**e**,**f**) Subsequent light pulses do not show substantial decrease of the depolarization peak, nor changes in the depolarization temporal dynamics (photoexcitation density, 343.9 mW/mm^2^; light stimuli duration: 100 ms; dark interval: 900 ms).

Light-induced changes to the plasma membrane potential have been further investigated by whole-cell recordings in current-clamp (*I* = 0) mode (Fig. 3). Data have been reported as relative variation of the membrane potential; no alteration in the resting membrane potential values were detected (see data reported in the SI section, Table S1, for cells cultured on P3HT and on control glass substrates). The comparison with non-transfected cells is once more instructive. In HEK-293 cells cultured onto P3HT samples and subjected to the same photoactivation densities, a cell membrane depolarization followed by a hyperpolarization signal is observed (see Figure S4 for a representative example of the variation of the membrane potential). The sign reversal occurs over an average temporal scale of few tens of milliseconds. Both these signals have been recently characterized and interpreted on the base of thermal phenomena, namely a variation of the membrane capacitance and the Nernst potential values, respectively^37^. Conversely, in HEK-293T cells the depolarization signal is recorded over much longer timescale, and it continuously increases over the whole considered stimuli duration of 100 ms (Fig. 3a). The initial, fast depolarization signal is still present (see a magnification in Fig. 3c, inset, and measurements acquired over a shorter timescale of 20 ms, Figure S5), but its dynamics is partially overlapped with a much stronger, longer activating signal. Even though it is not possible to completely disentangle the two signals, we estimated the build-up time constant in the order of 48 ± 0.3 ms, 105 ± 1.7 ms and 225 ± 22 ms for 343.9 mW/mm^2^, 202.4 mW/mm^2^ and 122.6 mW/mm^2^, respectively, by fitting the membrane potential variation with a single exponential curve within the temporal range 115 ÷ 200 ms (Fig. 3a, grey symbols). Moreover, the hyperpolarization observed in non-transfected cells is completely overwhelmed in HEK-293T cells by the longer-activating depolarization signal. Cells seeded on bare glass substrates and subjected to the same photostimulation protocols do not show any response, exception made for the highest photoexcitation density, where a small (<−0.7 mV) hyperpolarization signal is recorded (Fig. 3b and Figure S5). The average variation of the membrane potential shows a statistically significant difference respect to the value recorded on illuminated control samples (without the polymer) over the same temporal window (Student’s t-test, p < 0.001 in all cases). The maximum membrane potential variation linearly scales with the photoexcitation density (linear regression coefficient R^2^ = 0.98), Fig. 3d. Importantly, the photo-induced depolarization effect is highly repeatable and reliable. In fact, no significant changes are detected neither in the peak value nor in its temporal dynamics, as shown in Fig. 3e and f, respectively, for 16 subsequent light pulses.

### Identification of the TRPV1 response

**Figure 4:**
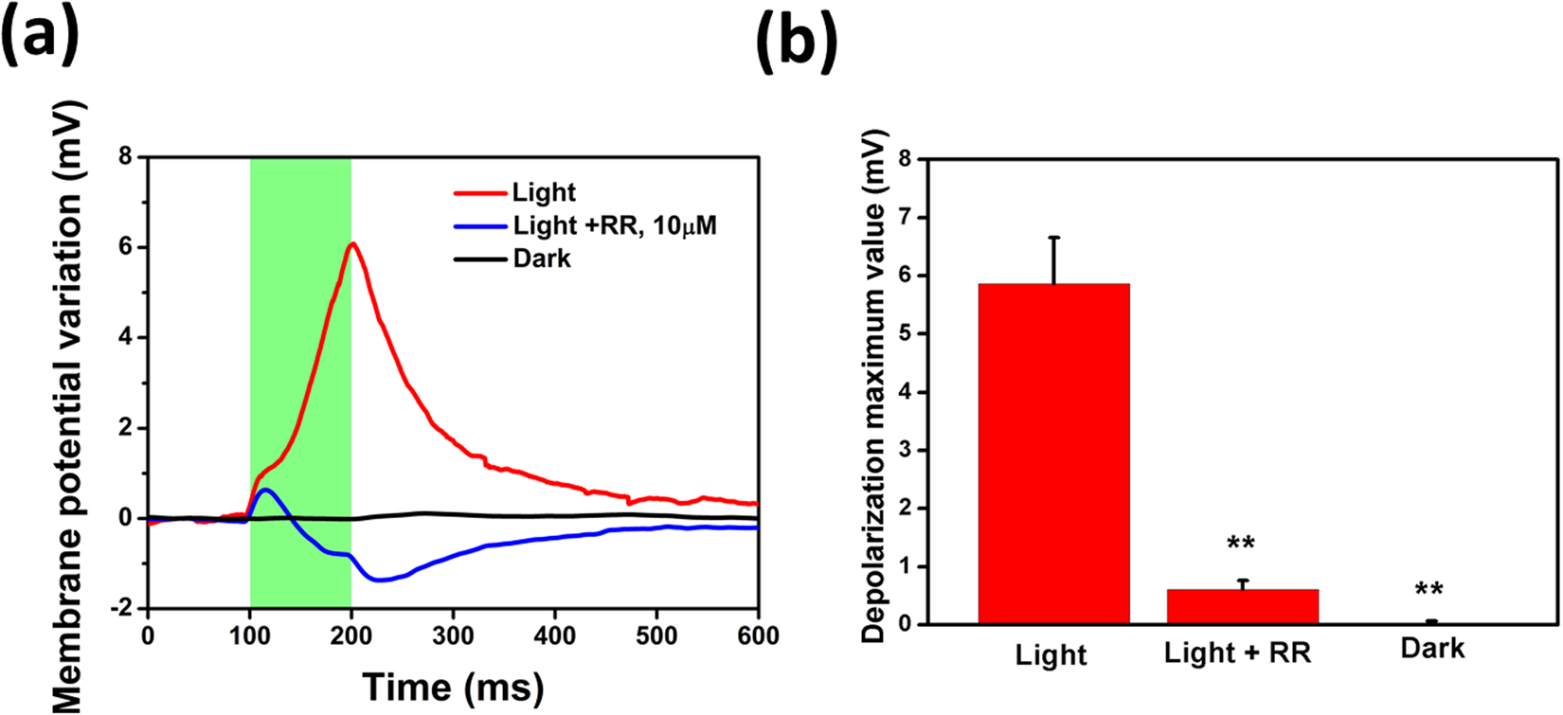
Administration of 10 µM RR completely suppresses the depolarization signal attributed to TRPV1 activation. (**a**) Representative membrane potential variation subsequently recorded in absence (red solid line) and presence of RR (blue solid line) on the same cell, in order to exclude cell-to-cell variability in RR exposure, upon 100-ms pulses stimulation (photoexcitation density, 343.91 mW/mm^2^). A representative control measurement in the dark (black solid line) is also shown for comparison. Every trace is obtained as the average of 40 consecutive sweeps. Data have been reported as relative membrane potential variation to better appreciate the light-induced effect; however, no significant changes were detected in the resting membrane potential values (mean ± SE, V_m_ = −12.5 ± 1.3 mV). (**b**) Mean ± MSE depolarization values in the three considered conditions, calculated over a population of n = 17 cells (Student’s t-test, **p < 0.001).

In order to corroborate the hypothesis that the current increase and the change in the membrane potential observed with the patch-clamp technique are effectively due to the photo-activation of the TRPV1 channel we treated the cells with the organometallic dye Ruthenium Red (RR). This compound, for decades, has been the only antagonist available to inhibit capsaicin-mediated responses, not only interacting with the ligand binding site, but also blocking its aqueous pore^38, 39^. Upon administration of 10 µM RR, the longer photo-activated depolarization is completely suppressed, and the hyperpolarization, observed in non-transfected cells lacking the TRPV1 channel, is now recordable (Fig. 4). For completeness, voltage-clamp recordings are shown in the SI section, Figure S6. The average reduction of the maximum depolarization, calculated as the average difference between the maximum depolarization recorded upon light before and after RR administration, is 88%. In order to exclude any variability issue among different cells, due to different exposure to RR, the variation has been evaluated on the same cell, prior and upon RR administration. The experiment has been repeated over a population of n = 17 cells.

**Figure 5:**
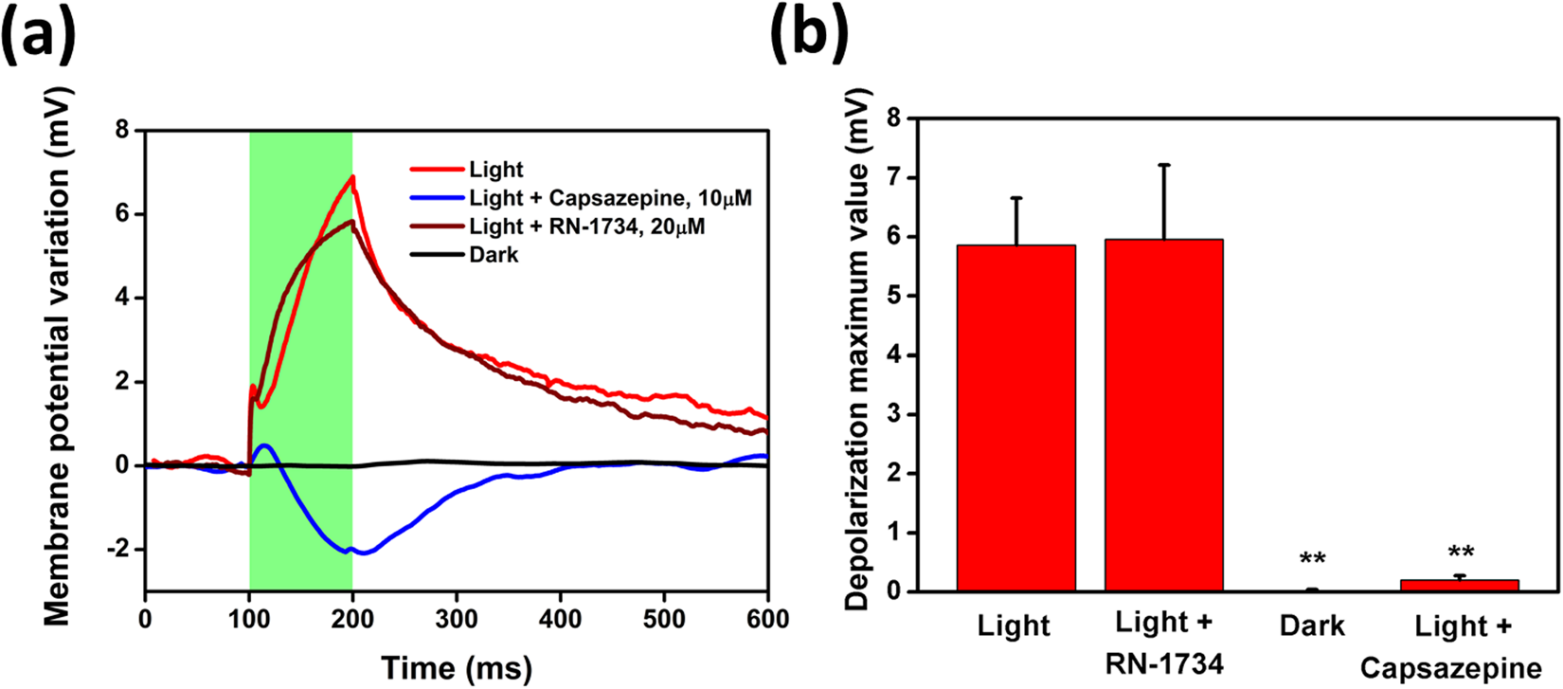
Photoinduced membrane potential variation in HEK-293T cells is fully blocked by the selective inhibitor Capsazepine, and it is not modified by the selective TRPV4 inhibitor RN-1734. (**a**) Representative membrane potential variation in HEK-293T cells seeded on P3HT polymer before and after administration of the TRPV1 selective blocker Capsazepine (10 µM) and TRPV4 selective blocker RN-1734 (20 µM), as compared to control experiments in dark. Every trace is the mean of 40 consecutive sweeps. 100 ms-light pulses, photoexcitation density 343.91 mW/mm^2^. Data have been reported as relative membrane potential variation to better appreciate the light-induced effect; however, no significant changes were detected in the resting membrane potential values (mean ± SE, V_m_ = −13 ± 1.9 mV and V_m_ = −13.5 ± 2.7 mV for cells treated with Capsazepine and RN-1734, respectively). All the experiments are performed at 24 °C. (**b**) mean ± MSE membrane potential variation values over a population of n = 10 cells for Capsazepine and n = 9 cells for RN-1734 (Student’s t-test, **p < 0.001).

RR is a non-competitive antagonist of TRPV1 and, based on recent studies, it can be classified as a general inhibitor of TRPV channels. Conversely, Capsazepine is a well-studied synthetic analogue of Capsaicin, which exhibits high TRPV1 selectivity as compared to other proteins of the TRPV family^38, 39^. We observe that, upon perfusion of 10 µM Capsazepine in the extracellular medium, the depolarization signal attributed to TRPV1 excitation is completely suppressed. As a further, positive control, we expose HEK-293T cells to the selective TRPV4-antagonist RN-1734 (20 µM)^40^. In this case, the photoinduced signal is unchanged (Fig. 5). For completeness, voltage-clamp recordings upon administration of capsazepine and RN-1734 are shown in the SI section, together with the calculated I-V characteristics (Figure S5).

### The photo-activation mechanism: the role of temperature and pH

Altogether, reported data fully demonstrate the possibility to use optical excitation mediated by thin films of conjugated polymers to efficiently activate TRPV1 channels.

**Figure 6:**
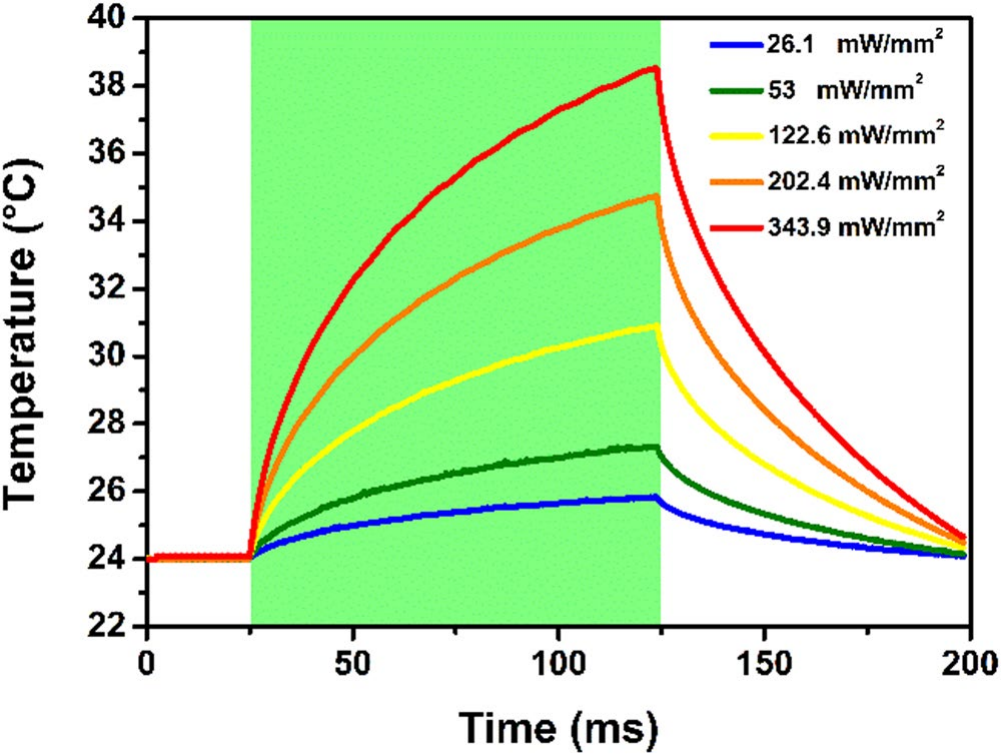
Temperature variation of the extracellular bath upon 100 ms photoexcitation. The temperature variation of the extracellular bath in the close proximity (~1 μm) of the polymer surface is measured as a function of time, at different photo-excitation densities.

TRPV1 is a multimodal channel, whose activation can be the result of a combination of multiple factors. However, this protein is primarily heat-sensitive, and a threshold temperature of 43 °C has been established in literature by a number of reports^23, 39, 41^. Thus, we first investigate the role of a locally confined, photothermal effect due to the heating of the bath in the close proximity of the polymer surface, as the most plausible origin of the observed channel activation. The temperature variation of the extracellular bath in the close proximity of the polymer absorbing layer has been evaluated by using the method of the calibrated pipette resistance^42^. Thanks to the wide optical absorption spectrum of the polymer, different excitation light sources in the visible range may be also used, leading to a different temperature increase (see Figure S7 for an example). The temporal profiles of the local heating upon green light excitation, at increasing light intensities, are depicted in Fig. 6 and Figure S8 for 100 ms- and 20 ms-pulses, respectively.

Upon illumination at the maximum photoexcitation density, the temperature increases by about 15 °C and 7 °C, for longer and shorter light pulses, respectively, remaining however below the reported activation threshold of 43 °C. On the other hand, we observe robust TRPV1 activation at photoexcitation densities in the order of 100 mW/mm^2^, corresponding to a temperature increase of about 6 °C and an absolute temperature of 30 °C, thus indicating that the temperature alone cannot be the origin of TRPV1 elicitation. The temperature dynamics can be fitted with a single exponential curve, obtaining a time constant **τ**_**T**_ around 50 ms for every considered photoexcitation density (Table 1). Interestingly, we observe that this value is in agreement with the channel activation time constant **τ**_**TRPV**_ only at the highest considered light intensity (343.9 mW/mm^2^), in physiological pH conditions. At lower power density, the activation of the channel occurs over longer timescales, as expected for sub-threshold activation (see Fig. 3a and Table 1).

**Table 1:** Time constant extracted by exponential fitting of the photo-induced temperature dynamics (τ_T_) and TRPV membrane potential variation (τ_TRPV_).

Light density (mW/mm^2^)	τ_T_ (ms)		τ_TRPV_ (ms)
122.6	45.3 ± 0.3		224.9 ± 22
202.4	47.2 ± 0.3		105 ± 1.7
343.9	48 ± 0.4	pH 5.0:	556 ± 51
		pH 6.0:	326 ± 23
		pH 7.4 (10 mM):	202 ± 5.3
		pH 7.4 (5 mM):	48 ± 0.3
		pH 8.0:	49 ± 1

Besides chemical agents like Capsaicin, other factors have been reported in literature for their capability to lower the TRPV1 activation temperature^8, 43^. In particular, it has been recently established that protons can sensitize TRPV1 receptors to capsaicin and heat. They both act to lower the channel threshold to levels at which the activation of temperature sensors is limited^8, 36, 44, 45^, and are able to activate the channel by themselves^46^. This is in line with a recent work by our group, in which we demonstrated that illumination of P3HT thin films immersed in water leads to a progressive acidification of the solution in the close proximity to the polymer surface, due to accumulation of photo-generated electrons, progressively charge balanced by the local formation of an electron-poor (acidic) layer of polarized water molecules^47^.

**Figure 7:**
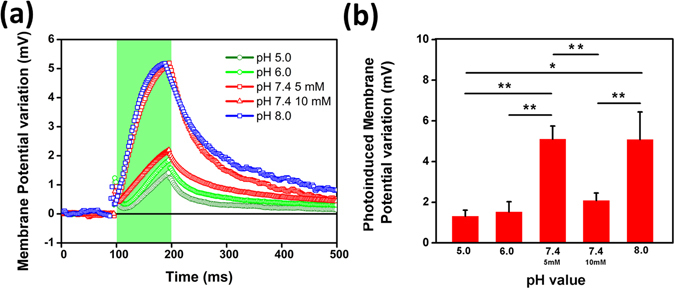
Effect of the extracellular pH. (**a**) Representative membrane potential variation upon photoactivation, at both acid and basic extracellular pH (100 ms light pulses, 343.91 mW/mm^2^ photoexcitation density). At physiological pH 7.4 two different buffer concentrations were used (5 mM and 10 mM, represented by square and triangles symbols, respectively). The experiments were performed at 24 °C. Every trace is the mean of 40 consecutive sweeps. (**b**) Membrane potential variation, averaged over n = 10 cells for each considered pH condition and reported as mean ± MSE values. *P < 0.05; **P < 0.001 (Student’s t-test).

In a further experiment, we examine the possibility to optically activate TRPV1 channel in different pH conditions of the extracellular medium (Fig. 7). Interestingly, the increase in the proton concentration turns out into a response less intense than the one observed at physiological and slightly basic pHs. This can be interpreted on the base of a pre-activation of the channel, before the light onset, due to the acid pH of the bath, as also supported by the observed shift of the resting membrane potential (V_m_) towards more positive values (V_m pH 6.0_ = −3.9 ± 1.2 mV; V_m pH 5.0_ = −3.4 ± 2 mV) compared to physiological pH (V_m pH7.4_ = −12.8 ± 4.4 mV). The photo-activated depolarization signal is possibly due to a further, supra activation of the channel. Conversely, in the case of basic pH, we can assume that the channel is completely closed before the light onset. Basic extracellular conditions usually hamper effective TRPV1 sensitization^46^. Upon photoexcitation, however, two local effects act concomitantly at the polymer surface, in the close proximity of the cell membrane, namely the acidification of the extracellular bath and the increase of the temperature. This makes it possible to activate the channel at temperatures below the theoretical threshold and despite the basic pH conditions of the bulk extracellular bath. Measurements under physiological pH at two different molar concentrations of the buffering agent confirm this picture: higher buffering capability (10 mM) leads to a reduced depolarization signal, being able to more efficiently counter-balance the effect of the pH variation optically induced by the organic polymer (Fig. 7). Time constant extracted by fitting the activation curves in different pH conditions with an exponential growth curve are in agreement with this picture. At acid pH, TRPV1 channels are efficiently opened already before the light onset, so that optical stimulation has only a limited, supra-threshold effect (longer dynamics). At physiological pH and upon 343.9 mW/mm^2^ photoexcitation density, activation dynamics are dominated by temperature effects, possibly coupled to a local, fast acidification of the extracellular environment.

## Conclusions

A novel method for spatially and temporally precise optical activation of TRPV channels is reported, based on the functional interfacing of the conjugated polymer P3HT, with HEK-293 cells stably expressing human TRPV1 channels. As evidenced by current- and voltage-clamp analysis, we obtain robust and reliable activation of the channel in physiological-like conditions (i.e., at the resting membrane potential), at temperatures well below the nominal activation threshold, and even in basic pH conditions. We identified two light-driven effects leading to channel activation, namely photo-thermal heating and a local pH variation. Both of them are locally confined at the interface between the conjugated polymer and the extracellular bath, thus offering enhanced spatial resolution as compared to other existing activation techniques, ideally even at the level of cell sub-compartments. Besides, existing methods to modulate the extracellular pH and to elicit TRPV1 activity are based on pharmacological assays, which most of the time lack temporal reversibility. Further engineering of the biopolymer interface will be needed to quantitatively disentangle the two mechanisms and further exploit this peculiar advantage. Moreover, at variance with other optical methods based on the use of infrared light sources, the use of visible light (only weakly absorbed by water) avoids detrimental, unlocalized and hardly controllable over-heating of the extracellular bath. Importantly, we did not observe any deleterious effect of the photostimulation protocols on cell viability, even at the highest photoexcitation densities. Here, we used green light excitation, properly matched to the P3HT absorption spectrum, but the demonstrated technique may be easily transferred to any other wavelength in the visible range simply by employing different active, organic semiconductors. Thus, also in virtue of its inherent simplicity, the reported method may be easily coupled to any electrophysiological set-up, equipped with standard fluorescence light sources, for *in vitro* studies. Based on the excellent biocompatibility shown by P3HT polymer thin films in *in vivo* studies, it is also conceivable to extend the reported technique to animal investigation.

In conclusion, we believe that the use of optically active polymer-based interfaces offers distinct advantages in the study of multimodal TRPV channels, and holds a huge potential in all related physiological applications.

## Materials and Methods

### Sample preparation

Regio-regular P3HT (99.995% purity, M_n_ 54.000–75.000 molecular weight) was purchased from Sigma Aldrich. All materials were used without any further purification. The samples for cell cultures were prepared by spin-coating on square 18 × 18 mm^2^ glass (VWR) substrates, carefully rinsed in subsequent ultrasonic baths of ultrapure water, acetone and isopropanol. P3HT solution was prepared in chlorobenzene at a final P3HT concentration of 20 g/l, and spin-coated on the cleaned substrates with a two-steps recipe: i) 3 s at 800 rpm, ii) 60 s at 1600 rpm. All films were thermally treated in an oven at 120 °C for 2 h for annealing and sterilization. To promote adhesion, samples were coated with fibronectin (from bovine plasma, Sigma Aldrich) at a concentration of 2 mg/ml in phosphate buffered saline (PBS) for at least 30 min at 37 °C and then rinsed with PBS.

### Cell culture maintenance and viability

All procedures were performed using immortalized cell lines, in accordance with the principle of the 3 R (Replacement, Reduction, Refinement) as established by the European Community Council (Directive 2012/63/EU of 22 September 2010) and were approved by the Italian Ministry of Health.

hTRPV1-HEK Recombinant Stable Cell Line was kindly provided by Dr. De Petrocellis (National Research Council, Biomolecular Chemistry Institute, CNR-ICB, Napoli, Italy). The cells were cultured in cell culture flasks containing Dulbecco’s modified Eagle’s medium (DMEM) added with 10% Fetal Bovine Serum (FBS), 100 U/ml Penicillin and 100 µg/ml Streptomycin. Culture flasks were maintained in a humidified incubator at 37 °C with 5% CO_2_. HEK-293 cells stably expressing human TRPV1 were cultured under 5% CO_2_ at 37 °C and, when at confluence, plated at a density of 15.000 cells/cm^2^ and cultured for 48 h on P3HT and glass samples. Proliferation was evaluated after 1, 2, 3 and 4 days *in vitro* with the MTT assay in three replicates. P3HT and glass samples were prepared as described above. HEK-293T were plated on each substrate at a concentration of 15,000 cells/cm^2^. For each time point, the culture medium was removed and replaced with fresh medium without serum and phenol red, supplemented with 0.5 mg/ml of MTT reagent; cells were re-incubated at 37 °C for 2 h. Culture medium was then removed and 1 ml of ethanol was added to dissolve formazan crystals. The absorbance of the solution (at 560 nm) was measured with Spark® 10 M Multimode Microplate Reader (Tecan Trading AG).

### Electrophysiology

Standard patch clamp recordings were performed using a Axopatch 200B (Axon Instruments) coupled to an inverted microscope (Nikon Eclipse Ti). HEK-293 cells (laboratory passage 20–22) were measured in whole-cell configuration with freshly pulled glass pipettes (3–6 MΩ), filled with the following intracellular solution [mM]: 12 KCl, 125 K-Gluconate, 1MgCl_2_, 0.1 CaCl_2_, 10 EGTA, 10 HEPES, 10 ATP-Na_2_. The extracellular solution contained [mM]: 135 NaCl, 5.4 KCl, 5 HEPES, 10 Glucose, 1.8 CaCl_2_, 1 MgCl_2_. For acidic pH solutions we used equimolar concentrations of 2-(N-morpholino)ethanesulfonic acid (MES) as buffering solution instead of HEPES. The temperature of the perfusate was controlled using a SC-20 dual in-line heater/cooler (Warner Instruments). Only single HEK-293 cells were selected for recordings. Acquisition was performed with pClamp-10 software (Axon Instruments). Membrane currents were low pass filtered at 2 kHz and digitized with a sampling rate of 10 kHz (Digidata 1440 A, Molecular Devices). Data were analyzed with Clampfit (Axon Instruments) and Origin 8.0 (OriginLab Corporation).

### Optical excitation

The light source for excitation of the polymer was provided by a LED system (Lumencor Spectra X) fibre-coupled to the fluorescence port of the microscope; the illuminated spot on the sample has an area of 0.23 mm^2^. Green emitting LED was used as light source, characterized by maximum emission wavelength at 544 nm and variable photoexcitation density, in the range 26 ÷ 344 mW/mm^2^, as measured at the output of the microscope objective (P_obj_). It is important to notice that, due to the high absorbance of the P3HT active layer, as well as to optical losses due to the petri-dish and the glass substrate, the actual optical density reaching the cell culture is reduced to 3.48 mW/mm^2^, 7.39 mW/mm^2^, 19.13 mW/mm^2^, 38.7 mW/mm^2^ and 86.96 mW/mm^2^, corresponding to impinging P_obj_ amounting at 26.1 mW/mm^2^, 53 mW/mm^2^, 122.6 mW/mm^2^, 202.4 mW/mm^2^, 343.9 mW/mm^2^, respectively. Light sources with different emission spectral range were also used, peaking in the blue (peak emission wavelength, 436 nm), in the cyan (peak emission wavelength, 485 nm) and in the red (peak emission wavelength, 629 nm). Photo-excitation density was the same in all considered cases (33 mW/mm^2^).

### Statistical analysis

Data are represented as mean ± MSE. The significance of differences between two groups was evaluated with unpaired Student’s t-test. P < 0.05 was considered statistically significant. *P < 0.05; **P < 0.001.

## Electronic supplementary material

Supporting Information

## References

[1] Camerino DC, Tricarico D, Desaphy JF (2007). Ion channel pharmacology. Neurotherapeutics.

[2] *Ion Channels: From Structure to Function*. Ed. by Kew, J. and Davies, C. Oxford University Press, New York (2010).

[3] Bagal SK (2013). Ion channels as therapeutic targets: a drug discovery perspective. J. Med. Chem..

[4] Pedersen SF, Owsianik G, Nilius B (2005). TRP channels: an overview. Cell Calcium.

[5] Nilius B, Owsianik G (2011). The transient receptor potential family of ion channels. Genome Biol..

[6] Venkatachalam K, Montell C (2007). TRP channels. Annu. Rev. Biochem..

[7] Gavva NR (2008). Body-temperature maintenance as the predominant function of the vanilloid receptor TRPV1. Trends Pharmacol. Sci..

[8] Tominaga M (1998). The cloned capsaicin receptor integrates multiple pain-producing stimuli. Neuron.

[9] Rigoni M (2003). Neurogenic responses mediated by vanilloid receptor-1 (TRPV1) are blocked by the high affinity antagonist, iodo-resiniferatoxin. Br. J. Pharmacol..

[10] Biggs JE (2008). Effect of SB-750364, a specific TRPV1 receptor antagonist, on injury-induced ectopic discharge in the lingual nerve. Neurosci. Lett..

[11] Li HB (2008). Antistress effect of TRPV1 channel on synaptic plasticity and spatial memory. Biol. Psychiatry.

[12] Medvedeva YV, Kim MS, Usachev YM (2008). Mechanisms of prolonged presynaptic Ca2+ signaling and glutamate release induced by TRPV1 activation in rat sensory neurons. J. Neurosci..

[13] Ho KW, Lambert WS, Calkins DJ (2014). Activation of the TRPV1 cation channel contributes to stress-induced astrocyte migration. Glia.

[14] Ward NJ, Ho KW, Lambert WS, Weitlauf C, Calkins DJ (2014). Absence of transient receptor potential vanilloid-1 accelerates stress-induced axonopathy in the optic projection. J. Neurosci..

[15] Weitlauf C (2014). Short-term increases in transient receptor potential vanilloid-1 mediate stress-induced enhancement of neuronal excitation. J. Neurosci..

[16] Sappington RM (2015). Activation of transient receptor potential vanilloid-1 (TRPV1) influences how retinal ganglion cell neurons respond to pressure-related stress. Channels.

[17] Gomtsyan A (2005). Novel transient receptor potential vanilloid 1 receptor antagonists for the treatment of pain: structure-activity relationships for ureas with quinoline, isoquinoline, quinazoline, phthalazine, quinoxaline, and cinnoline moieties. J Med Chem.

[18] Messeguer A, Planells-Cases R, Ferrer-Montiel A (2006). Physiology and pharmacology of the vanilloid receptor. Curr. Neuropharmacol..

[19] Szallasi A, Cortright DN, Blum CA, Eid SR (2007). The vanilloid receptor TRPV1: 10 years from channel cloning to antagonist proof-of-concept. Nat. Rev. Drug Discov..

[20] Broad LM, Keding SJ, Blanco MJ (2008). Recent progress in the development of selective TRPV1 antagonists for pain. Curr. Top. Med. Chem..

[21] Nilius B, Szallasi A (2014). Transient receptor potential channels as drug targets: from the science of basic research to the art of medicine. Pharmacol. Rev..

[22] Gunthorpe MJ, Harries MH, Prinjha RK, Davis JB, Randall A (2000). Voltage- and time-dependent properties of the recombinant rat vanilloid receptor (rVR1). J. Physiol..

[23] Caterina MJ (1997). The capsaicin receptor: a heat-activated ion channel in the pain pathway. Nature.

[24] Yang F (2015). Structural mechanism underlying capsaicin binding and activation of the TRPV1 ion channel. Nat. Chem. Biol..

[25] Siemens J (2006). Spider toxins activate the capsaicin receptor to produce inflammatory pain. Nature.

[26] Jordt SE, Tominaga M, Julius D (2000). Acid potentiation of the capsaicin receptor determined by a key extracellular site. Proc. Natl. Acad. Sci..

[27] Ross RA (2003). Anandamide and vanilloid TRPV1 receptors. Br. J. Pharmacol..

[28] Frank JA (2015). Photoswitchable fatty acids enable optical control of TRPV1. Nat. Commun..

[29] Lyu Y, Xie C, Chechetka SA, Miyako E, Pu K (2016). Semiconducting Polymer Nanobioconjugates for Targeted Photothermal Activation of Neurons. J. Am. Chem. Soc..

[30] Barbarella G, Melucci M, Sotgiu G (2005). The Versatile Thiophene: An Overview of Recent Research on Thiophene-based Materials. Adv. Mater..

[31] Ghezzi D (2011). A hybrid bioorganic interface for neuronal photoactivation. Nat. Commun..

[32] Antognazza MR (2016). Characterization of a Polymer-Based, Fully Organic Prosthesis for Implantation into the Subretinal Space of the Rat. Adv. Healthc. Mater..

[33] Vaquero Morata S (2016). Conjugated polymers for the optical control of the electrical activity of living cells. J. Mater. Chem. B.

[34] Tortiglione C (2017). Semiconducting polymers are light nanotransducers in eyeless animals. Science Advances..

[35] Feyen P (2016). Light-evoked hyperpolarization and silencing of neurons by conjugated polymers. Sci. Rep..

[36] Hui K, Liu B, Qin F (2003). Capsaicin activation of the pain receptor, VR1: multiple open states from both partial and full binding. Biophys. J..

[37] Martino N (2015). Photothermal cellular stimulation in functional bio-polymer interfaces. Sci. Rep..

[38] Vriens J, Appendino G, Nilius B (2009). Pharmacology of vanilloid transient receptor potential cation channels. Mol. Pharmacol..

[39] Brito R, Sheth S, Mukherjea D, Rybak LP, Ramkumar V (2014). TRPV1: A Potential Drug Target for Treating Various Diseases. Cells.

[40] Dragoni S (2015). A functional transient receptor potential vanilloid 4 (TRPV4) channel is expressed in human endothelial progenitor cells. J. Cell Physiol..

[41] Voets T (2004). The principle of temperature-dependent gating in cold- and heat-sensitive TRP channels. Nature.

[42] Yao J, Liu B, Qin F (2009). Rapid temperature jump by infrared diode laser irradiation for patch-clamp studies. Biophys. J..

[43] Guenther S, Reeh PW, Kress M (1999). Rises in [Ca2+]i mediate capsaicin- and proton-induced heat sensitization of rat primary nociceptive neurons. Eur. J. Neurosci..

[44] Reeh PW, Kress M (2001). Molecular physiology of proton transduction in nociceptors. Curr. Opin. Pharmacol..

[45] Ryu S, Liu B, Qin F (2003). Low pH potentiates both capsaicin binding and channel gating of VR1 receptors. J. Gen. Physiol..

[46] Aneiros E (2011). The biophysical and molecular basis of TRPV1 proton gating. EMBO J..

[47] Mosconi, E. *et al*. Surface Polarization Drives Photoinduced Charge Separation at the P3HT/Water Interface. *ACS Energy Lett*. 454–463 (2016).

