# Supplementary material for: Conjugated polymers mediate effective activation of the Mammalian Ion Channel Transient Receptor Potential Vanilloid 1
Source: Sci Rep. 2017 Aug 16;7:8477. doi: 10.1038/s41598-017-08541-6 (PMC5559550; doi:10.1038/s41598-017-08541-6)
Supplement: Supplementary file 1 — Supporting Information [file 41598_2017_8541_MOESM1_ESM.pdf]

Conjugated polymers mediate effective activation of the Mammalian Ion Channel Transient Receptor Potential Vanilloid 1

F. Lodola[1], N. Martino[1,#], G. Tullii[1,2], G. Lanzani[1,2], M. R. Antognazza[1*].

[1]*Center for Nano Science and Technology, IIT@PoliMi, via Pascoli 70/3, 20133, Milano, Italy*
[2]*Politecnico di Milano, Dipartimento di Fisica, Piazza L. Da Vinci 32, 20133, Milano, Italy*

[#]*Current address: Wellman Center for Photomedicine, Massachusetts General Hospital and Harvard Medical School, Cambridge, Massachusetts 02139, USA*

*Corresponding author: Mariarosa.antognazza@iit.it

SUPPLEMENTARY INFORMATION

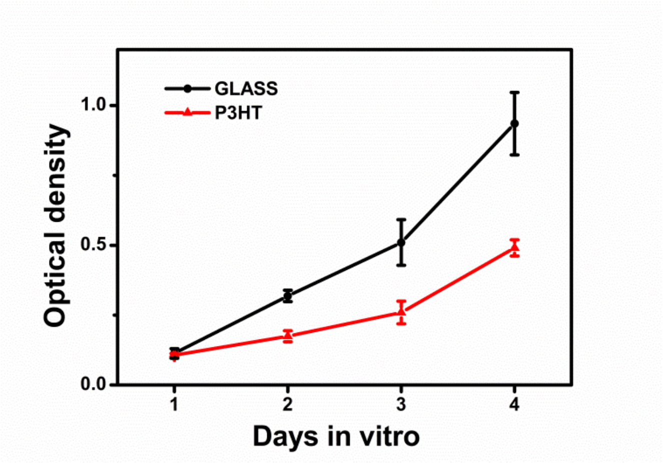

Figure S1 | HEK-293T cells viability. MTT assay for HEK-293T cells proliferation up to 4 days in vitro. Cells cultured on top of P3HT polymer surface (red symbols) show good viability properties, even though their proliferation rate is slightly reduced as compared to control glass substrates (black symbols).

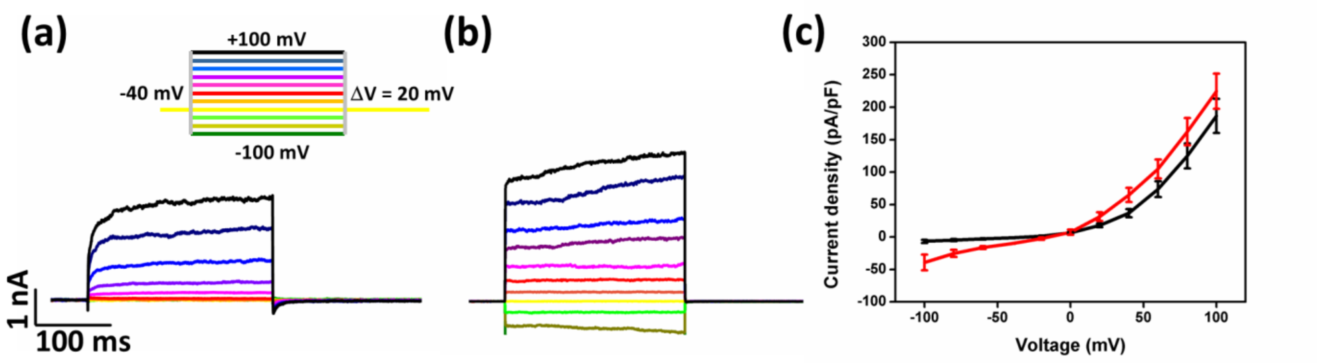

Figure S2 | Capsaicin-induced TRPV1 activation. Representative whole-cell current traces recorded in absence **(a)** and in presence of 50 nM Capsaicin **(b)** from HEK-293T cells, in response to voltage steps from -100 to +100 mV from an holding potential of -40 mV (protocol in the inset). The cells were seeded on glass, the experiments performed at 24°C. **(c)** Current-voltage characteristics before and after capsaicin administration (black and red symbols, respectively). Values are obtained as mean ± MSE, n=10 cells.

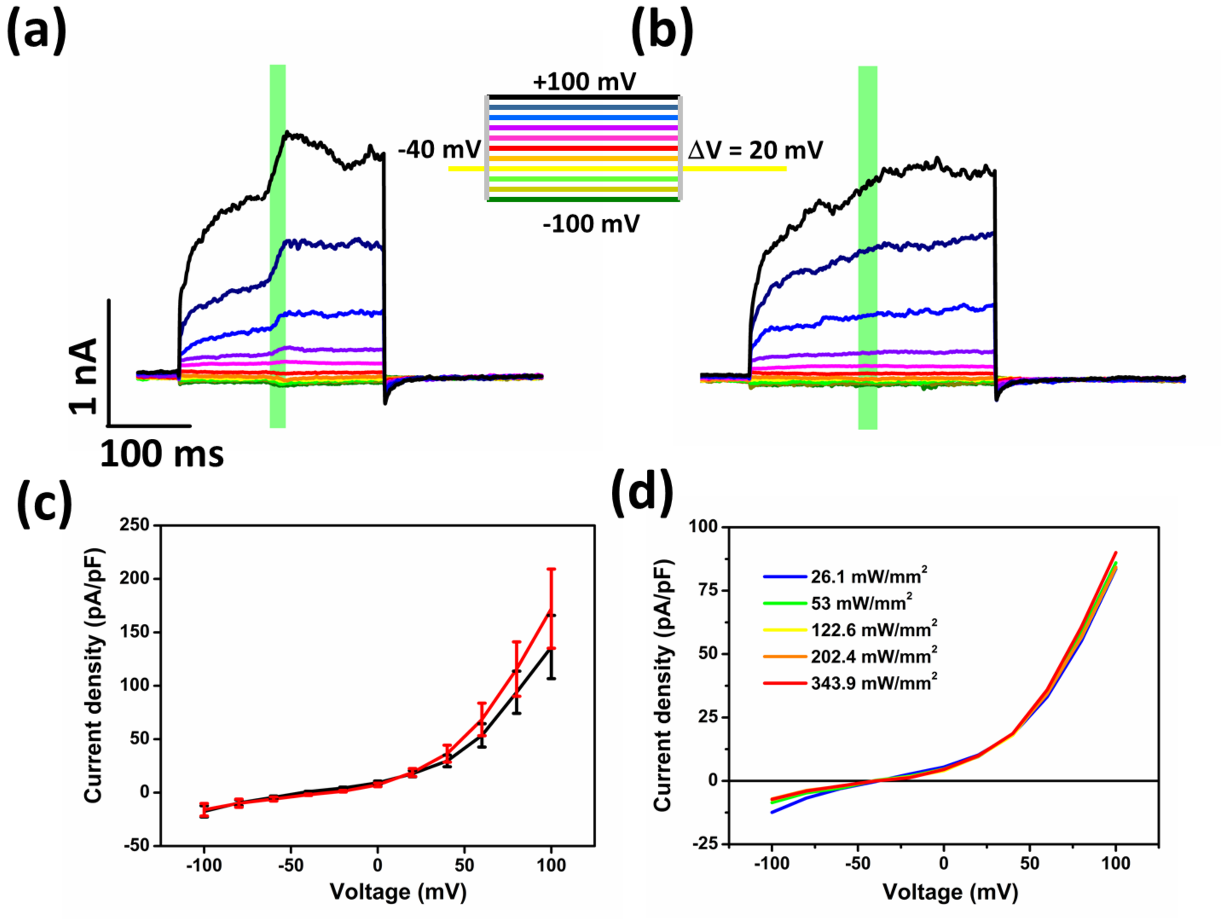

Figure S3 | Current increase in HEK-293T cells upon 20 ms light pulses. Representative whole-cell current traces recorded from HEK-293T cells in response to voltage steps from -100 mV to +100 mV from an holding potential of -40 mV. The cells were seeded on P3HT **(a)** and glass **(b)**, and photostimulated with 20 ms pulses of light (photoexcitation density, 202.39 mW/mm^2). **(c)** Current-voltage characteristics acquired in cells immediately before the light onset (black line) and before the light offset (red line). Data have been calculated as an average over 5 ms-wide temporal windows. **(d)** Current voltage characteristics recorded under different photoexcitation densities, within the range 26.1 mW/mm^2 – 343.9 mW/mm^2. Data have been calculated as the average values recorded during the whole temporal window of the illumination protocol (green rectangle in panel (a)).

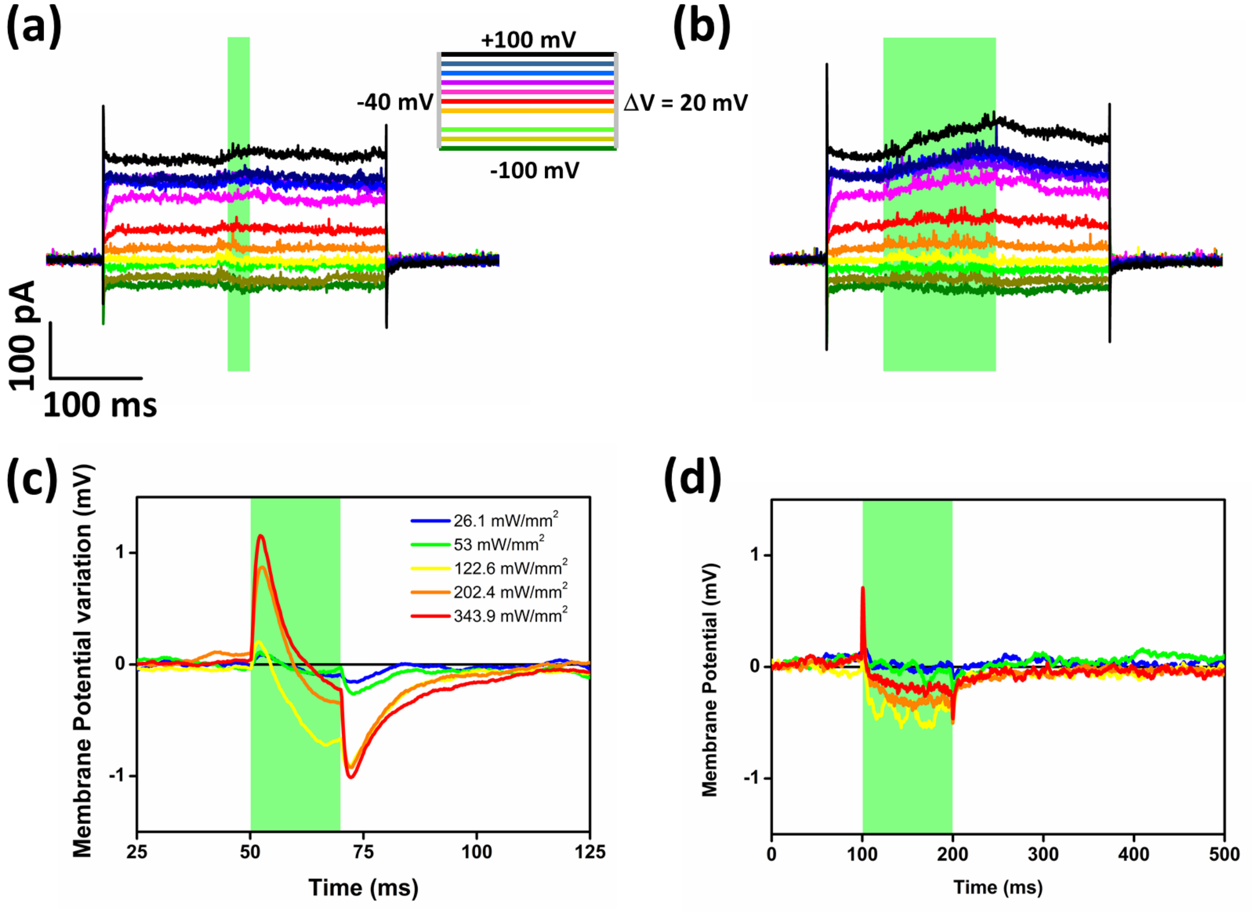

Figure S4 | Optical stimulation of non-transfected HEK-293 cells. Voltage clamp **(a, b)** and current clamp (I = 0) **(c, d)** measurements in non-transfected HEK-293 cells, subjected to 20 ms (left panels) and 100 ms (right panels) light pulses, photoexcitation density, 343.9 mW/mm^2. Both the transient depolarization signal and the hyperpolarization observed for longer stimuli are due to the heating of the extracellular bath, mediated by the polymer photoexcitation. In particular, the first effect is due to an increase of the membrane capacitance, while the second one is the consequence of a variation in the membrane reversal potential. See Ref. [1] for a more detailed discussion.

[1] N. Martino et al., Martino, N., Feyen P., Porro, M., Bossio, C., Zucchetti, E., Ghezzi, D., Benfenati, F., Lanzani, G., Antognazza, M. R. Photothermal cellular stimulation in functional bio-polymer interfaces. *Sci. Rep.* **5**, 8911 (2015).

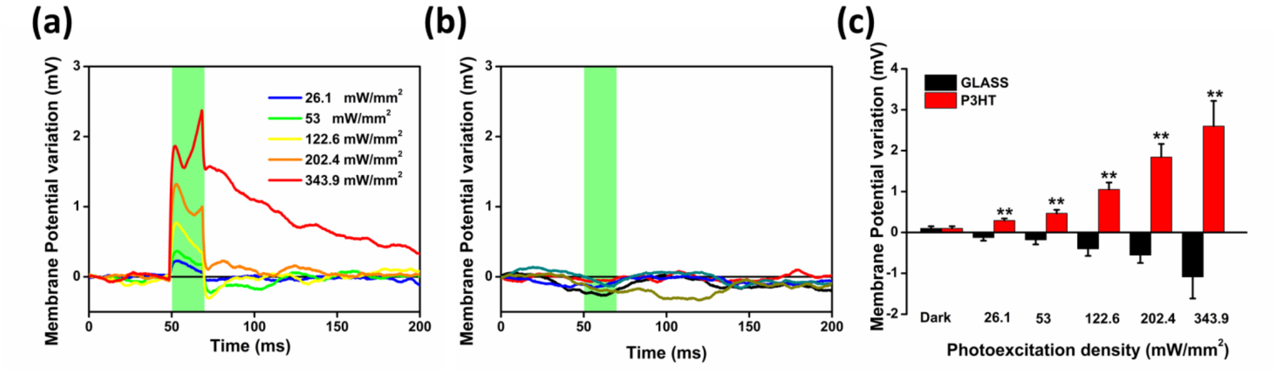

Figure S5 | Membrane potential variation in HEK-293T cells upon 20 ms light pulses, seeded on P3HT **(a)** and on glass **(b).** Photoexcitation density range, 26.1 ÷ 343.9 mW/mm^2. The experiments are performed at 24°C. Every trace is the mean of 40 consecutive sweeps. **(c)** Membrane potential variation as a function of the photoexcitation density, on P3HT and on glass control samples. Values are reported as mean ± MSE, n=10 cells each condition. (**: $p < 0.001$, Student's t-test).

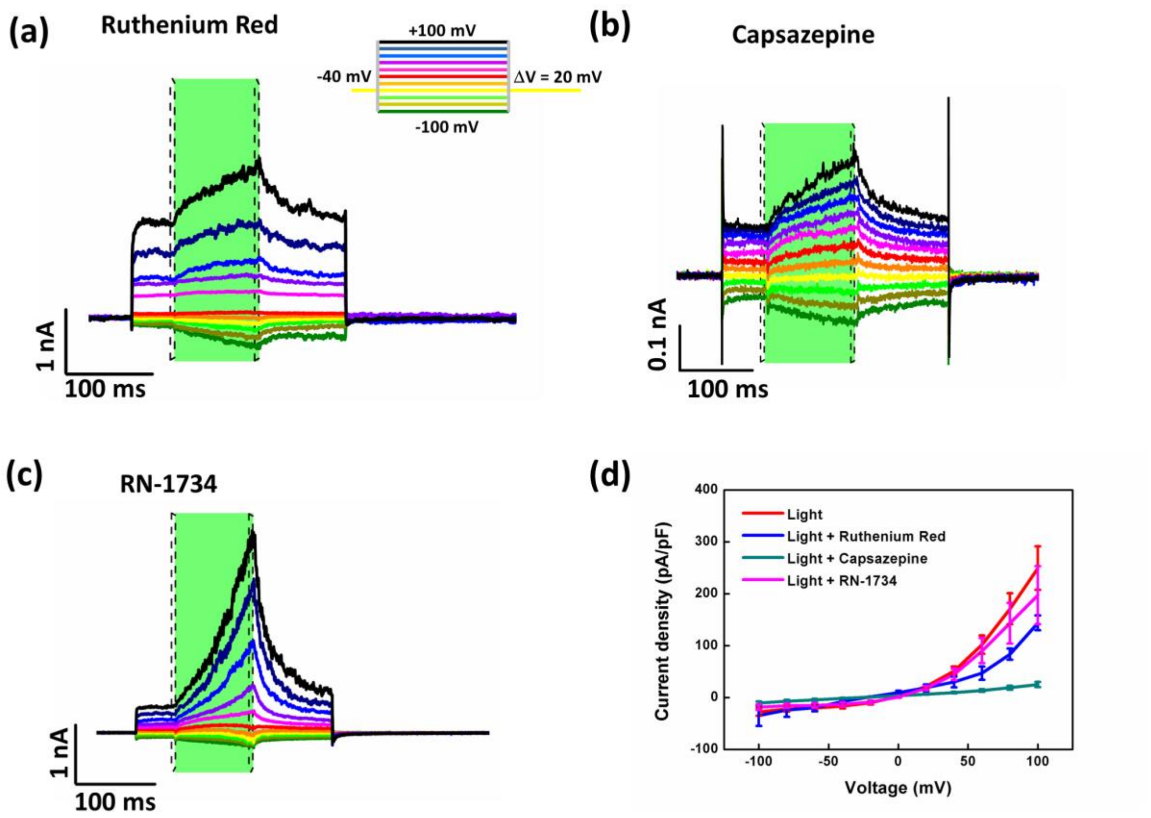

Figure S6 | Voltage-clamp recordings upon photoexcitation and pharmacological treatment. Representative whole-cell current traces in response to voltage steps from -100 mV to +100 mV. Holding potential, -40 mV (protocol in the inset). Photoexcitation is represented by the green shaded area (photoexcitation density, 343.9 mW/mm^2; pulse duration, 100 ms). Every trace is the mean of 40 consecutive sweeps. **(a)** Non-selective TRPV inhibitor Ruthenium Red, 10 μM molar concentration. **(b)** Selective TRPV1 inhibitor Capsazepine, 10 μM molar concentration. **(c)** selective TRPV4-antagonist RN-1734, 20 μM molar concentration. **(d)** Current–voltage characteristics acquired in cells immediately before the light onset (black line) and upon light (color solid lines). Data have been calculated as an average over the 5 ms-wide temporal windows represented in panels (a), (b) and (c) as dashed rectangles. Values are reported as mean ± MSE, n=6 cells for each condition. The experiments are performed at 24°C.

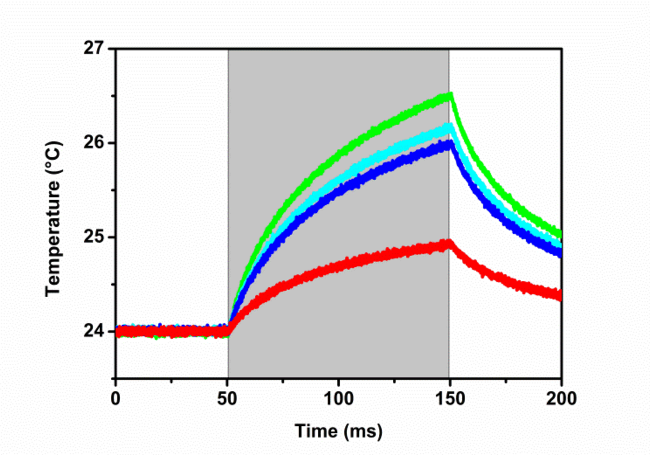

Figure S7 | Temperature variation of the extracellular bath upon 100 ms photoexcitation with different light sources. 4 different LEDs have been used, with emission spectrum peaking at 436 nm (blue), 485 nm (cyan), 544 nm (green) and 629 nm (red). Device thickness, light incidence direction and photo-excitation density (33 mW/mm^2, as measured at the corresponding peak emission wavelength) were the same in all considered cases.

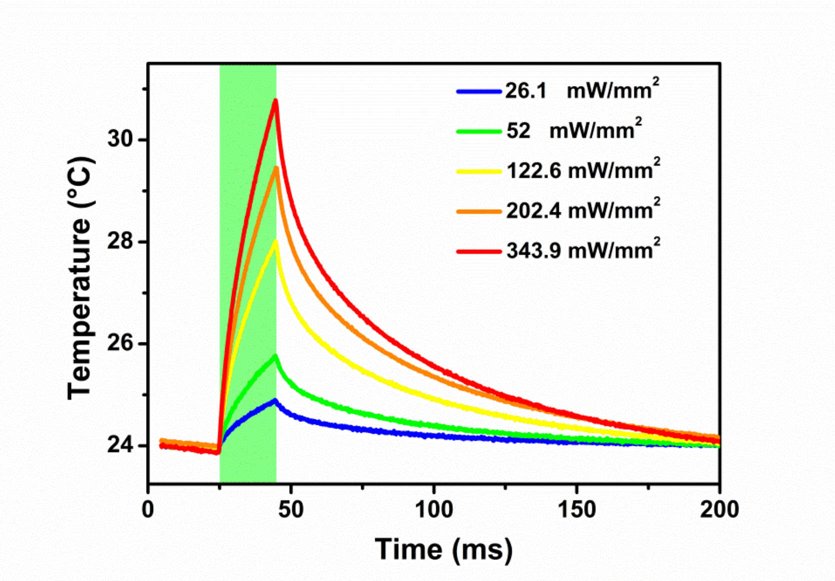

Figure S8 | Temperature variation of the extracellular bath upon 20 ms photoexcitation. Increase in the absolute temperature of the extracellular bath in close proximity to the P3HT surface upon 20 ms light pulses at photoexcitation densities in the range 26.1 ÷ 343.9 mW/mm^2.

	P3HT		Glass	
Photoexcitation Density	V_m	ΔV	V_m	ΔV
mW/mm^2	mV	mV	mV	mV
26,1	-14.3 ± 2.70	0.34 ± 0.09	-13.2 ± 3.46	-0.18 ± 0.07
53	-14.9 ± 1.72	0.86 ± 0.34	-15.2 ± 3.21	-0.26 ± 0.16
122,6	-15.1 ± 3.02	1.99 ± 0.54	-15.3 ± 2.60	-0.35 ± 0.23
202,4	-13.3 ± 1.92	3.59 ± 0.34	-14.0 ± 2.14	-0.68 ± 0.22
343,9	-14.0 ± 2.03	5.10 ± 0.63	-15.0 ± 1.80	-1.40 ± 0.62

Table S1 | Resting membrane potential (V_m) and membrane potential variation (ΔV) values of cells cultured on top of P3HT and control glass substrates, subjected to optical stimulation at different photoexcitation densities. Data are reported as mean ± SE values calculated over the same statistical set of cells reported in Figure 3a (P3HT) and Figure 3b (Glass) of the main article.
